# Factors associated with indoor smoking at home by adults across Korea: a focus on socioeconomic status

**DOI:** 10.4178/epih.e2020067

**Published:** 2020-10-28

**Authors:** Bomgyeol Kim, Yejin Lee, Young Dae Kwon, Tae Hyun Kim, Jin Won Noh

**Affiliations:** 1 Department of Public Health, Yonsei University, Seoul, Korea; 2 Department of Healthcare Management, Eulji University, Seongnam, Korea; 3 Department of Humanities and Social Medicine, College of Medicine and Catholic Institute for Healthcare Management, The Catholic University of Korea, Seoul, Korea; 4 Department of Healthcare Management, Graduate School of Public Health, Yonsei University, Seoul, Korea; 5 Department of Health Administration, Dankook University, Cheonan, Korea

**Keywords:** Smoke-free policy, Smoking prevention, Family health, Community Health Survey

## Abstract

**OBJECTIVES:**

Secondhand smoke is an issue that cannot be ignored due to its various negative effects. Especially, secondhand smoke inside the household is an area where health policy must pay attention as it can affect all age groups. This study aims to identify the factors associated with smoking inside the household focusing on socioeconomic status in Korea.

**METHODS:**

We used data from the Community Health Survey of 2017 and a total of 33,462 participated in the study. Data were analyzed through IBM SPSS version 25.0 to conduct binary logistic regression analysis.

**RESULTS:**

Results indicated that indoor smoking had a significant association with socioeconomic status. This association was more marked in those participants who had low household income or those with elementary school education level or less. Furthermore, the study indicates that when the smoker is a woman, older, has higher stress, and is a heavier smoker, the probability of her smoking inside the house is higher.

**CONCLUSIONS:**

Based on the results, it is meaningful that this study has found the factors of smoking inside household. The result identify the factors associated with indoor smoking at home, and it can be used as baseline data for developing new smoking cessation policies.

## INTRODUCTION

Passive (or secondhand) smoking refers to a condition in which a non-smoker is exposed to harmful substances in cigarettes by inhaling the smoker’s cigarette smoke [1,2]. With the widespread awareness of the dangers of passive smoking, the issue of third-hand smoke in the home was raised recently. Third-hand smoke means that harmful substances generated during smoking are absorbed into the hair, body, clothing, furniture, etc. of the smoker and then transmitted to others through the process of being discharged back into the air [3]. Particularly, nicotine and tar are easily adsorbed into wallpaper or furniture due to their stickiness. When they interact with other components in the indoor air, they turn into carcinogenic substances and negatively affect the human body [4,5]. The amount of nicotine in the house exposed to smoking for a long time may be higher than that from direct smoking of a single cigarette, and it is even more dangerous as the toxicity of cigarettes accumulates and continues to affect the members of the household [6].

Damage from passive smoking includes not only simple discomfort from cigarette smoke, but also various diseases. Lung cancer, coronary heart disease, and stroke have been reported as consequences of passive smoking [7]. Particularly, children who are exposed to passive smoking for more than an hour each day have more than three times higher incidence of attention deficit and hyperactivity disorder (ADHD) than unexposed children, and they are more susceptible to middle ear infection, respiratory symptoms, and lung function damage [8]. As such, the number of diseases caused by passive smoking continues to increase, resulting in more deaths. More than 890,000 people die each year from passive smoking in the world [9,10]. Passive smoking at home affects not only smokers, but also their families, and has a negative effect in terms of increased medical expenses. Therefore, the World Health Organization (WHO) and other governments are working to reduce smoking rates [11].

Since joining the Framework Convention on Tobacco Control in 2005 to reduce smoking rates, Korea has also promoted policies to implement the convention, such as expanding non-smoking areas, raising cigarette taxes, and introducing warning texts and pictures on cigarette packages [12]. Despite these efforts, the advent of heated tobacco products is leading to an increase in the number of people who smoke heated tobacco products in their homes. A survey of 2,000 heated tobacco products smokers conducted by the Korean Society for Research on Nicotine and Tobacco revealed that 77.5% of those who switched from regular cigarettes to heated tobacco products smoked the heated tobacco products in their homes [13]. Despite the risk of exposure to passive smoking being higher than direct smoking, it is very difficult to regulate passive smoking at home. In Korea and other countries, participating the Framework Convention on Tobacco Control, there are few policies that regulate passive smoking at home. Smoking cessation at home should be performed voluntarily; the government cannot enforce it [14]. Therefore, it is necessary to identify and manage the factors related to indoor smoking at home.

A review of previous studies indicated that various factors such as gender, age, place of residence, education level, household income, number of children, number of smokers in the household, and smoking cessation rules in the household are associated with indoor smoking at home [15-17]. Until now, studies related to passive smoking in the household mainly focused on exposure to passive smoking in the household for non-smokers [18]. Although there was a study by Jeong [19] on smokers, the subject of smoking, the study was limited as it only investigated citizens residing in Seoul. Therefore, this study aims to identify the factors related to indoor smoking at home by adults across Korea, focusing on socioeconomic factors, based on the Korea Community Health Survey (CHS) data that can represent Korean adults. The results of this study are intended to be used as a basis for smoking cessation policies targeting indoor smokers who smoke in their homes.

## MATERIALS AND METHODS

### Materials and respondents

This study is conducted based on data from the 2017 CHS which is conducted to produce regional health statistics for establishing and evaluating a local healthcare plan, and it has been conducted every year since 2008 under the supervision of the Korea Centers for Disease Control and Prevention [20]. After selecting sample points in the administrative units of tong, ban, and ri using probability proportional to size sampling, the households were selected using the systematic extraction method. A 1:1 computer-assisted personal interviewing was conducted on adults aged 19 years and older living in residential houses of the selected administrative units [20]. The raw data can be obtained after filling in a data use plan on the CHS website (https://chs.cdc.go.kr/chs/index.do).

**Figure 1.**
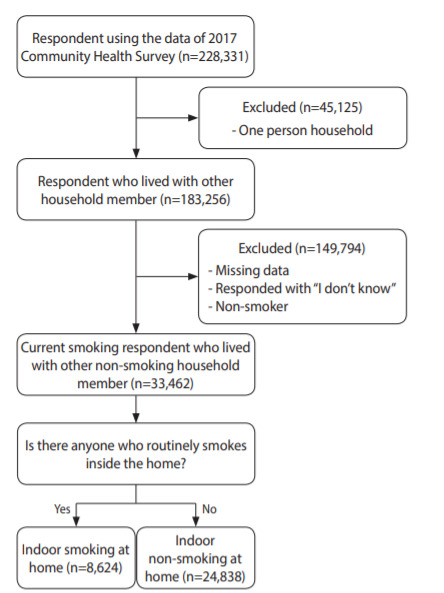
Flow chart of research subjects.

Of the 228,381 persons in the raw data of 2017 CHS, 183,256 persons from households consisted of two or more members were selected, excluding 45,125 persons from single-person households. Respondents who did not respond to the variables included in the analysis of this study or responded “I don’t know” were excluded. Since this study was conducted on smokers, 33,462 persons were sampled excluding non-smokers. Finally, indoor smoking at home was defined as one or more non-smoking members of the household answered “yes” to the question, “Is there anyone who routinely smokes inside the home?” There were 24,838 smokers who did not smoke indoors and 8,624 smokers who smoked indoors (Figure 1).

### Variables

#### Dependent variable

Indoor smoking status was used as the dependent variable. It was classified as indoor non-smoking at home if non-smoking members of the household answered “no” to the question, “Is there anyone who routinely smokes inside the home?” [19,21]. Indoor smoking at home was defined as one or more non-smoking members of the household answered “yes” to the question, “Is there anyone who routinely smokes inside the home?”

#### Independent variables

Factors related to indoor smoking at home were selected and classified into demographic factors, socioeconomic factors, and smoking-related factors [15-18,22-25]. Demographic factors included gender, age, marital status, and number of children under 19 years in the household. Age was classified into “19-44 years old,” “45-64 years old,” and “65 years old or older,” based on the life cycle in the CHS guidelines [20]. Marital status was classified into “single,” “other (divorced/widowed/separated),” and “married” [22]. Using a continuous variable, the number of children under 19 years in the household was classified into “0,” “1,” “2,” and “3 or more.”

Socioeconomic factors included education level, economic activity, and monthly household income. Education level was classified into “elementary school or lower,” “middle school,” “high school,” and “college or higher.” Economic activity status was classified into “yes” or “no” as answers to the question, “Have you worked more than an hour to generate income or worked as an unpaid family worker for more than 18 hours in the past week?” Monthly household income was classified into “less than 3 million Korean won (KRW),” “3 to 4 million KRW,” “4 to 5 million KRW,” and “more than 5 million KRW” [23].

Health-related factors included self-rated health and self-rated stress. For self-rated health, based on the answers depending on the usual physical condition, “very good” and “good” were classified into “good;” “normal” into “normal;” and “bad” and “very bad” into “bad” [24]. For self-rated stress, based on the answers depending on the level of stress felt in day to day life, “very stressed” and “stressed” were classified into “high,” and “a little stressed” and “not stressed” into “low” [25].

Smoking-related factors included the number of cigarettes per day, intention to quit smoking, exposure to quit smoking campaigns, and education to quit smoking. The number of cigarettes per day was classified into “1 to 9 cigarettes,” “10 to 19 cigarettes,” “20 to 39 cigarettes,” and “40 or more cigarettes” [25]. Intention to quit smoking was classified into “1 month,” “6 months,” “someday,” and “no plan” as answers to the question, “Do you have any plans to quit smoking in the future?” Exposure to quit smoking campaigns was classified as “yes” for having seen or heard public service advertisements (TV, radio, posters, leaflets, etc.) about smoking cessation in the past year. Education to quit smoking was classified as “yes” for having received smoking prevention or cessation education in the past year.

### Statistical analysis

Frequency analysis was conducted to identify the general characteristics of the study participants. A chi-square test was performed to determine the indoor smoking status at home according to the characteristics of the study participants. Binary logistic regression analysis was conducted to identify factors related to indoor smoking at home. IBM SPSS version 25.0 (IBM Corp., Armonk, NY, USA) was used for analysis, and the significance level was set to p-value< 0.05.

### Ethics statement

The Institutional Review Board at the Korea Centers for Disease Control and Prevention approved the study protocol (2013-06EXP-01-3C). This study was conducted using CHS data, ethics approval was not required.

## RESULTS

### Descriptive analysis of the general characteristics of the respondents

The total number of participants was 33,462. Table 1 presents the participants’ general characteristics. There were 24,838 participants (74.2%) who did not smoke indoors at home and 8,624 (25.8%) who smoked indoors at home. Significant correlation with indoor smoking at home was found in all variables including gender, age, marital status, number of children under 19 years in the household, education level, household income, self-rated health, self-rated stress, number of cigarettes per day, intention to quit smoking, exposure to quit smoking campaigns, and education to quit smoking.

### Logistic regression analysis with smoke-free indoor as the dependent variable

Binary logistic regression analysis was performed to identify factors related to indoor smoking at home. Table 2 presents the analysis results. Smokers were less likely to smoke indoors at home if they had received education to quit smoking (odd ratio [OR], 1.11; 95% confidence interval [CI], 1.04 to 1.20); if they were married (divorced/widowed/separated OR, 1.35; 95% CI, 1.24 to 1.47; married OR, 1.38; 95% CI, 1.22 to 1.56, respectively); if there were more children under 19 years in the household (1 children OR, 1.44; 95% CI, 1.33 to 1.56; 2 children OR, 1.71; 95% CI, 1.56 to 1.87 or more children OR, 1.58; 95% CI, 1.35 to 1.85, respectively); if they had a college or higher education compared to those with elementary school or lower level of education (OR, 1.47; 95% CI, 1.33 to 1.63); if the average monthly household income was higher (KRW 4 to 5 million OR, 1.09; 95% CI, 1.00 to 1.18; more than KRW 5 million OR, 1.20; 95% CI, 1.11 to 1.29, respectively); and if their self-rated health was good (normal OR, 1.17; 95% CI, 1.06 to 1.26: good OR, 1.27; 95% CI, 1.17 to 1.38). Smokers were more likely to smoke indoors at home if they were women (OR, 0.47; 95% CI, 0.43 to 0.52); if they were older (45 to 64 years old OR, 0.75; 0.70 to 0.81; 65 years old or older OR, 0.76; 95% CI, 0.68 to 0.84, respectively); if they were under a high level of stress (OR, 0.90; 95% CI, 0.85 to 0.95); if they smoked more cigarettes per day (10 to 19 cigarettes OR, 0.67; 95% CI, 0.62 to 0.72; 20 to 39 cigarettes OR, 0.48; 95% CI, 0.44 to 0.52; 40 or more cigarettes OR, 0.29; 95% CI, 0.25 to 0.35, respectively); and if they had no plan to quit smoking (OR, 0.70; 95% CI, 0.62 to 0.79).

## DISCUSSION

This study identified the factors related to indoor smoking at home based on the 2017 CHS data. The study results revealed that smokers were more likely to smoke indoors at home if they were women, were older, were under a high level of stress, smoked more cigarettes per day, and had no plan to quit smoking. Smokers were less likely to smoke indoors at home if they were married, had more children under 19 in the household, had a college or higher education compared to those with elementary school or lower level of education, had a higher average monthly household income, had good self-rated health, had been exposed to quit smoking campaigns, and had received education to quit smoking.

Regarding socioeconomic factors, we found that smokers were less likely to smoke indoors at home if the average monthly household income was higher and if they had a college or higher education compared to those with elementary school or lower level of education, which is in line with previous studies demonstrating that indoor non-smoking status was associated with high education level and monthly income [17,18]. This can be explained by the results of studies suggesting that parents of low-income households have low knowledge of the dangers of passive smoking, with relatively lukewarm attitudes and fewer actions taken against passive smoking [26]. Thus, indoor smoking is reported to be affected by socioeconomic factors such as income and education level, various policy approaches are needed to increase the willingness of people with low socioeconomic status to cease smoking indoors [27,28].

Regarding demographic factors, smokers were more likely to smoke indoors at home if they were women. This could be explained by the fact that the main smoking places for woman smokers were indoor toilets and indoor verandas at home [29-31]. Increased use of electronic cigarettes in women and increased indoor use of electronic cigarettes that hardly smell with their smoke converted to water vapor may also be the contributing factors [14,30]. Smokers were more likely to smoke indoors at home if they were older as the elderly were less aware of the risks associated with indoor smoking at home than younger people, resulting in a higher chance of passive smoking in the household [31]. Smokers were less likely to smoke indoors at home if their marital status was either married or other and if there were more household members under 19 years in the household. It appeared when the marital status was married or other, there was a high possibility of having children and refraining from smoking indoors at home [23]. Since there was a high possibility of household members under 19 years being their own children, it could be explained by the results of previous studies suggesting that people with young children were less likely to smoke indoors at home [23,32,33]. This suggested that the adverse effects of passive smoking on children and adolescents were well known, which was considered to be a positive factor as it might motivate smokers to quit smoking in the future. Regarding health-related factors, smokers were less likely to smoke indoors at home if their self-rated health was good, which could be explained by the results of previous studies that self-rated health was associated with choosing positive health behavior [34,35]. Those who rated their subjective health as good seemed to avoid smoking, as it negatively impacted their health, and they were less likely to smoke indoors. In contrast, smokers were more likely to smoke indoors under a higher level of self-rated stress. It appeared that those under high stress were more likely to engage in smoking with a higher chance of smoking indoors [35]. Regarding smoking-related factors, smokers were more likely to smoke indoors at home if they smoked more cigarettes per day, which aligns with the results of previous studies that smokers were less likely to smoke indoors when they smoked less frequently [23,36,37]. This also aligned with previous studies reporting a higher success rate of smoking cessation associated with less smoking and lower nicotine dependence, suggesting that the less smokers smoked, the less likely other household members were to be exposed to passive smoking at home [38]. Smokers were more likely to smoke indoors at home if they had no plan to quit smoking. This could be explained by previous studies demonstrating that the more willing the smoker was to quit, the lower the chance of smoking indoors [39]. Smokers were less likely to smoke indoors at home if they received education to quit smoking, which aligns with the results of previous studies that smoking cessation education positively induces smoking cessation by increasing the willingness of participants to quit smoking. Smoking cessation education motivates the participants to quit smoking [40]. Since 2005, public health centers (239 as of June 2019) and national smoking cessation support centers have provided representative education programs including the smoking prevention education project for households and the smoking cessation education for workplaces [41]. The smoking prevention education project for households provides the booklet “The First Step in Parental Education to Protect Our Children from Smoking” for early childhood smoking prevention education, and the smoking cessation education for workplaces is provided by the Korea Health Promotion Institute in collaboration with the local community (public health centers and regional smoking cessation support centers) for creating a non-smoking environment in workplaces [41,42]. Thus, there are various smoking cessation education programs, but there are no differentiated educational programs in consideration of the restrictions on temporal accessibility, size, and characteristics of the workplace. In particular, small-scale workplaces remain as blind spots for health promotion projects [40]. Therefore, it would be necessary to produce a variety of smoking cessation education materials and expand visiting smoking cessation support services by providing smoking cessation classes in residential areas as well as workplaces.

The limitations of this study are as follows. First, since this study only analyzed the data for one year, only the relationships between the variables were identified, and it was difficult to determine causal relationships. Second, due to the limitation of the data being secondary data, various factors such as environmental characteristics like the number of smoking population in the household excluding the study participants and the presence of smoking cessation rules in the household, and psychological characteristics of smokers were not considered. Moreover, if there were several smokers in the household, the cases where the smokers other than the participants of this study causing passive smoking in non-smoking household members may have been included in the analysis.

Despite these limitations, this study identified various factors related to indoor smoking at home and confirmed the association of indoor smoking at home with socioeconomic factors such as income and education level. This study is meaningful in that it used CHS data that is representative of Korean adults to investigate the factors related to indoor smoking of adults at home, focusing on socioeconomic factors. The results of this study are expected to be used as a basis for smoking cessation policies to reduce indoor smoking at home.

## Figures and Tables

**Table 1.** General characteristics of study participants (n=33,462)

Variables	At home		χ^2^
	Not indoor smoking	Indoor smoking	
Gender			232.446^***^
Men	23,288 (75.3)	7,652 (24.7)	
Women	1,550 (61.5)	972 (38.5)	
Age (yr)			330.033^***^
19-44	11,114 (79.2)	2,914 (20.8)	
45-64	10,489 (71.3)	4,222 (28.7)	
≥65	3,235 (68.5)	1,488 (31.5)	
Marital status			53.935^***^
Single	4,665 (72.1)	1,808 (27.9)	
Divorced/widowed/separated	18,536 (75.2)	6,104 (24.8)	
Married	1,637 (69.7)	712 (30.3)	
Child in household (<19 yr)			596.651^***^
0	15,259 (70.0)	6,528 (30.0)	
1	4,327 (80.0)	1,080 (20.0)	
2	4,240 (84.2)	797 (15.8)	
≥3	1,012 (82.2)	219 (17.8)	
Education level			589.099^***^
≤Elementary school	2,651 (66.1)	1,357 (33.9)	
Middle school	2,256 (65.4)	1,193 (34.6)	
High school	10,656 (73.0)	3,944 (27.0)	
≥College	9,275 (81.3)	2,130 (18.7)	
Economic activity			140.384^***^
No	3,932 (68.0)	1,848 (32.0)	
Yes	20,906 (75.5)	6,776 (24.5)	
Monthly household income (10^4^ KRW)			74.521^***^
<300	10,653 (71.9)	4,158 (28.1)	
300-400	4,920 (75.6)	1,585 (24.4)	
400-500	3,792 (76.4)	1,173 (23.6)	
≥500	5,473 (76.2)	1,708 (23.8)	
Self-rated health			219.742^***^
Bad	3,203 (66.2)	1,638 (33.8)	
Normal	11,106 (74.3)	3,842 (25.7)	
Good	10,529 (77.0)	3,144 (23.0)	
Self-rated stress			8.841^**^
Low	17,190 (74.7)	5,820 (25.3)	
High	7,648 (73.2)	2,804 (26.8)	
No. of cigarettes per day			483.258^***^
1-9	5,176 (80.9)	1,225 (19.1)	
10-19	10,306 (76.8)	3,119 (23.2)	
20-39	8,946 (69.3)	3,956 (30.7)	
≥40	410 (55.9)	324 (44.1)	
Intention to quit smoking			314.544^***^
1 mo	1,642 (80.3)	404 (19.7)	
6 mo	2,663 (78.9)	712 (21.1)	
Someday	12,782 (76.4)	3,942 (23.6)	
No plan	7,751 (68.5)	3,566 (31.5)	
Exposure to quit smoking campaigns			8.043^**^
No	1,522 (71.6)	603 (28.4)	
Yes	23,316 (74.4)	8,021 (25.6)	
Education to quit smoking			5.859^*^
No	20,997 (74.0)	7,384 (26.0)	
Yes	3,841 (75.6)	1,240 (24.4)	

Values are presented as number (%). KRW, Korean won.

* p<0.05,

** p<0.01,

*** p<0.001.

**Table 2.** Results of binominal logistic regression analysis

Variables	OR (95% CI)
Gender	
Men	1.00 (reference)
Women	0.47 (0.43, 0.52)^***^
Age (yr)	
19-44	1.00 (reference)
45-64	0.75 (0.70, 0.81)^***^
≥65	0.76 (0.68, 0.84)^***^
Marital status	
Single	1.00 (reference)
Divorced/widowed/separated	1.35 (1.24, 1.47)^***^
Married	1.38 (1.22, 1.56)^***^
Child in household (<19 yr)	
0	1.00 (reference)
1	1.44 (1.33, 1.56)^***^
2	1.71 (1.56, 1.87)^***^
≥3	1.58 (1.35, 1.85)^***^
Education	
≤Elementary school	1.00 (reference)
Middle school	0.92 (0.83, 1.01)
High school	1.09 (0.99, 1.19)
≥College	1.47 (1.33, 1.63)^***^
Economic activity	
No	1.00 (reference)
Yes	1.16 (1.08, 1.25)^***^
Monthly household income (104 KRW)	
<300	1.00 (reference)
300-400	1.05 (0.96, 1.15)
400-500	1.09 (1.00, 1.18)^*^
≥500	1.20 (1.11, 1.29)^***^
Self-rated health	
Bad	1.00 (reference)
Normal	1.17 (1.08, 1.26)^***^
Good	1.27 (1.17, 1.38)^***^
Self-rated stress	
Low	1.00 (reference)
High	0.90 (0.85, 0.95)^***^
No. of cigarettes per day	
1-9	1.00 (reference)
10-19	0.67 (0.62, 0.72)^***^
20-39	0.48 (0.44, 0.52)^***^
≥40	0.29 (0.25, 0.35)^***^
Intention to quit smoking	
1 mo	1.00 (reference)
6 mo	0.92 (0.80, 1.05)
Someday	0.91 (0.81, 1.02)
No plan	0.70 (0.62, 0.79)^***^
Exposure to quit smoking campaigns	
No	1.00 (reference)
Yes	1.01 (0.91, 1.12)
Education to quit smoking	
No	1.00 (reference)
Yes	1.11 (1.04, 1.20)^**^

OR, odds ratio; CI, confidence interval; KRW, Korean won.

* p<0.05,

** p<0.01,

*** p<0.001.

## SUPPLEMENTARY MATERIALS

Korean version is available at http://www.e-epih.org/.
