# Supplementary material for: Factors associated with indoor smoking at home by adults across Korea: a focus on socioeconomic status
Source: Epidemiol Health. 2020 Oct 28;42:e2020067. doi: 10.4178/epih.e2020067 (PMC8137374; doi:10.4178/epih.e2020067)
Supplement: Supplementary file 1 [file epih-42-e2020067-suppl.docx]

**Factors Associated with Indoor Smoking at Home: Focusing on Characteristics of Smokers**

가정 실내 흡연 관련 요인: 흡연자의 특성을 중심으로

Bomgyeol Kim^1^, Yejin Lee^2^, Young Dae Kwon^3^, Tae Hyun Kim^4^, Jin-Won Noh^5^

^1^Department of Public Health, Yonsei University, Seoul, Korea,

^2^Department of Healthcare Management, Eulji University, Seongnam, Korea,

^3^Department of Humanities and Social Medicine, The Catholic University, Seoul, Korea,

^4^Department of Healthcare Management, Graduate School of Public Health, Yonsei University, Seoul, Korea,

^5^Department of Health Administration, Dankook University, Cheonan, Korea

**Correspondence**: Jin-Won Noh

Department of Health Administration, Dankook University, Cheonan, Korea

E-mail: jinwon.noh@gmail.com

**Abstract**

OBJECTIVES: Secondhand Smoke is an issue that cannot be ignored due to various negative effects. Especially, secondhand smoke inside household is an area where health policy must pay attention as it can affect all age groups. This study is to figure out the factors associated with smoking inside household focusing on socioeconomic status in South Korea. METHODS: We used data from Community Health Survey of 2017 and a total of 33,462 participated in the study. It was analyzed through IBM SPSS Statistics 25.0 to conduct binary logistic regression analysis. RESULTS: The results showed that indoor smoking had a significant associated with socioeconomic status. This association was more marked in those who had low household income, or those with elementary school education level or less. Furthermore, the study indicates, when the smoker is a woman, older, has higher stress and is heavier smoker, the probability of her smoking inside is higher. CONCLUSIONS: Based on the results of this study, it is meaningful that the research has found the factors of smoking inside household. This result indicates the factors associated with indoor smoking at home, and it can be used as base data for making smoking cessation policy.

Key Words: Smoke-Free Policy, Smoking Prevention, Family Health, Community Health Survey

**INTRODUCTION**

간접흡연(Passive smoking, Second-hand smoking)은 비흡연자가 흡연자의 담배연기를 마심으로써 담배의 유해한 물질에 노출되는 상태를 의미한다 [1-2]. 간접흡연의 위험성에 대한 인식의 확산과 더불어 최근에는 가정에서의 3차 간접흡연 문제가 제기되었다. 3차 흡연(Third-hand smoking)이란 흡연 시 발생하는 유해물질이 흡연자의 머리카락이나 몸, 의류, 가구 등에 흡착되었다가 다시 공기 중으로 배출되는 과정을 통해 다른 사람에게 전달되는 것을 말한다 [3]. 특히 끈적이는 성질을 갖고 있는 니코틴과 타르는 벽지 또는 가구에 쉽게 흡착되고, 실내 공기 중의 다른 성분과 만나면서 발암성 물질로 변해 인체에 부정적 영향을 미친다 [4,5]. 흡연이 장기간 이루어졌던 집 실내의 니코틴 양은 담배 한 개비를 직접 흡연했을 때의 양보다 많을 수 있으며, 담배의 독성이 누적되면서 지속적으로 가족들에게 영향을 끼칠 수 있다는 점에서 더욱 위험하다 [6].

간접흡연으로 인한 피해는 담배연기로 인한 단순 불쾌감뿐만 아니라 다양한 질환으로 이어진다. 간접흡연으로 인한 질환은 대표적으로 폐암, 관상동맥심질환, 뇌졸중이 보고되었다 [7]. 특히 어린이는 간접흡연에 매일 1시간 이상 노출될 경우 노출 경험이 없는 아이에 비해 주의력 결핍 및 과잉행동장애(ADHD) 발생률이 3배 이상 높으며, 중이질환, 호흡계 증상, 폐기능 손상이 발생하는 것으로 알려졌다 [8]. 이처럼 간접흡연으로 야기되는 질병은 계속 증가하고 있으며, 그로 인한 사망 또한 증가하고 있다. 매년 전 세계에서 89만 명 이상이 간접흡연에 의해 사망하고 있으며 [9,10], 특히 가정에서의 간접흡연은 흡연자뿐만 아니라 가족들에게도 영향을 미치며 의료비 증가 측면에서도 부정적인 영향을 미치고 있어 세계보건기구(World Health Organization)와 각국 정부는 흡연율을 감소시키기 위해 노력하고 있다 [11].

우리나라 또한 흡연율을 감소시키기 위해 2005년 담배규제기본협약(Framework Convention on Tobacco Control) 가입 이후 금연구역 확대, 담배세 인상, 담뱃갑 경고 문구 및 경고 그림 도입 등 협약을 이행하기 위한 정책을 추진해왔다 [12]. 이러한 노력에도 불구하고 궐련형 전자담배의 등장으로 집 안에서도 궐련형 전자담배를 피우는 사람이 증가하고 있다. 대한금연학회에서 궐련형 전자담배 흡연자 2000명을 대상으로 설문한 결과, 일반담배에서 궐련형 전자담배로 바꾼 사람 중 77.5%가 집 안에서 궐련형 전자담배를 펴본 것으로 나타났다 [13]. 이처럼 직접흡연보다 간접흡연에 노출될 위험이 증가함에도 불구하고 가정에서의 간접흡연을 규제하는 것은 매우 어렵다. 우리나라를 비롯한 담배규제기본협약의 당사국에서 가정 내 간접흡연을 규제하는 정책은 찾기 어렵다. 가정에서의 금연 행위는 가정에서 자발적으로 받아들여야 하는 것이지 정부가 강제로 집행할 수 없기 때문이다 [14]. 따라서 흡연자의 가정 실내 흡연 관련 요인을 파악하여 관리할 필요가 있다.

선행연구를 살펴보면 성별, 연령, 거주지, 교육수준, 가구소득, 자녀 수, 가정 내 흡연자수, 가정 내 금연규칙 다양한 요인이 가정 내 흡연과 관련이 있는 것으로 나타난다 [15-17]. 지금까지 가정 내 간접흡연 관련 연구는 비흡연자를 대상으로 한 가정 내 간접흡연 노출에 연구가 주를 이루었고 [18], 흡연의 주체인 흡연자를 대상으로 한 연구는 정혜민(2016) 연구가 있었으나 서울시민으로만 한정된 연구이었다 [19]. 따라서 이 연구는 한국 성인을 대표할 수 있는 지역사회건강조사를 활용하여 사회경제적 요인을 중심으로 전국 성인의 가정 실내 흡연 관련 요인을 파악하고자 한다. 이를 통해 가정 실내 흡연자를 대상으로 하는 금연 정책을 위한 근거자료로 활용되고자 한다.

**MATERIALS AND METHODS**

**Materials and respondents**

이 연구는 2017 지역사회건강조사(Community Health Survey, CHS) 자료를 활용하였다. 지역사회건강조사는 지역보건의료계획을 수립 및 평가를 위한 지역건강통계 생산을 목적으로 시행되는 조사로, 질병관리본부가 주관하여 2008년부터 매년 시행되고 있다 [20]. 통∙반, 리 단위로 확률비례추출법을 통해 표본지점을 선정한 후 계통추출법을 이용해 조사가구를 선정하였으며, 통∙반, 리의 주거용 주택에 거주하는 19세 이상 성인을 대상으로 1:1 전자설문조사(CAPI: Computer Assisted Personal Interviewing) 방식으로 조사되었다 [20]. 지역사회건강조사 홈페이지를 통해 자료이용계획서 작성 후 원시자료를 획득할 수 있다.

원시자료 228,381명 중 2017 지역사회건강조사에서 1인 가구 45,125명을 제외하여 2인 가구 이상인 183,256명을 추출하였다. 또한 본 연구 분석에 포함된 변수에 응답을 하지 않았거나 모름으로 응답한 응답자를 제외하였으며, 흡연자를 대상으로 연구를 진행하였기 때문에 비흡연자를 제외하여 33,462명을 추출하였다. 최종적으로 ‘가정의 실내에서 일상적으로 담배를 피우는 분이 있습니까?’ 질문에 비흡연 동거가구원 1명 이상이 있음으로 응답하였을 경우를 기준으로 가정 실내 흡연을 정의하였다. 실내 비흡연의 경우 24,838명, 실내 흡연의 경우 8,624명이었다.

<Figure 1 삽입>

**Variables**

*Dependent variable*

종속변수는 가정 실내 흡연 여부를 사용하였다. 흡연자와 거주하는 비흡연자 가구원이 ‘가정의 실내에서 일상적으로 담배를 피우는 분이 있습니까?’ 질문에 가정 실내 흡연자가 없다고 응답하였을 때 실내 비흡연으로 판단하였다 [19,21]. 실내 흡연은 비흡연 동거 가구원이 1명이라도 가정 내 흡연자가 있다고 응답한 경우로 하였다.

*Independent variables*

이 연구에서는 가정 실내 흡연과 관련이 있는 요인들을 선정하여 인구학적 요인, 사회경제적 요인, 흡연 관련 요인으로 구분하였다 [15-18,22-25]. 인구학적 요인으로 성별, 연령, 결혼상태, 19세 미만 가구원수를 선정하였다. 연령은 지역사회건강조사 지침서에서 고시하는 생애주기인 ‘19-44세’, ‘45-64세’, ‘65세 이상’으로 구분하였다 [20]. 결혼상태는 ‘미혼’, ‘기타(이혼/사별/별거)’, ‘기혼’으로 구분하였다 [22]. 19세 미만 가구원수는 연속형 변수를 사용하여 ‘0명’, ‘1명’, ‘2명’, ‘3명 이상’으로 구분하였다.

사회경제적 요인으로 교육수준, 경제활동 여부, 월평균 가구소득을 선정하였다. 교육수준은 ‘초등학교 졸업 이하’, ‘중학교 졸업’, ‘고등학교 졸업’, ‘대학 졸업 이상’으로 구분하였다. 경제활동 여부는 ‘최근 1주일 동안 수입을 목적으로 1시간 이상 일을 하거나, 18시간 이상 무급 가족 종사자로 일하신 적이 있습니까?’라는 설문에 응답한 그대로 ‘예’, ‘아니오’로 구분하였다. 월평균 가구소득은 ‘300만원 미만’, ‘300만원 이상 400만원 미만’, ‘400만원 이상 500만원 미만’, ‘500만원 이상’으로 구분하였다 [23].

건강관련 요인으로는 주관적 건강상태, 주관적 스트레스 수준을 선정하였다. 주관적 건강수준은 평소 본인의 건강상태에 따라 ‘매우 좋음’, ‘좋음’, ‘보통’, ‘나쁨’, ‘매우 나쁨’으로 응답한 것을 ‘매우 좋음’과 ‘좋음’을 ‘좋음’, ‘보통’은 ‘보통’, ‘나쁨’과 ‘매우 나쁨’은 ‘나쁨’으로 분류하였다 [24]. 주관적 스트레스 수준은 평소 본인의 일상생활 중에 느끼는 스트레스의 정도에 따라 ‘대단히 많이 느낀다’와 ‘많이 느끼는 편이다’는 ‘높은 스트레스 수준’, ‘조금 느끼는 편이다’와 ‘거의 느끼지 않는다’는 ‘낮은 스트레스 수준’으로 분류하였다 [25].

흡연 관련 요인으로 하루 흡연량, 금연계획 여부, 금연캠페인 노출 경험, 금연교육 경험을 선정하였다. 하루 흡연량은 ‘1-9개비’, ‘10-19개비’, ‘20-39개비’, ‘40개비 이상’으로 구분하였다 [25]. 금연계획 여부는 “앞으로 담배를 끊을 계획이 있습니까?”라는 설문에 응답한 그대로 ‘1개월 내 금연’, ‘6개월 내 금연’, ‘언젠가는 금연’, ‘금연계획 없음’으로 사용하였다. 금연캠페인 노출 경험은 최근 1년 동안 금연에 대한 공익광고(TV, 라디오, 포스터, 리플릿 등)를 보거나 들어본 적이 있는 경우 ‘있음’으로 구분하였다. 금연교육 경험은 최근 1년 동안 흡연예방 또는 금연 교육을 받은 적이 있는 경우 ‘있음’으로 구분하였다.

Analysis method

연구 대상자의 일반적 특성을 알아보기 위해 빈도분석을 실시하였으며, 연구 대상자의 특성에 따른 가정 실내 흡연 여부를 알아보기 위하여 교차분석(Chi-square test)을 시행하였다. 가정 실내 흡연 관련 요인을 파악하고자 이분형 로지스틱회귀분석(binary logistic regression analysis)을 실시하였다. 분석은 IBM SPSS Statistics 25.0 프로그램을 활용하였으며, 유의수준은 p<0.05로 하였다.

**RESULTS**

**Descriptive analysis of the general characteristics of the respondents**

이 연구의 전체 대상자는 총 33,462명이었으며, 일반적 특성은 Table 1과 같다. 가정 실내 비흡연이 24,838명(74.2%), 가정 실내 흡연이 8,624명(25.8%)이었다. 각 요인별 가정 실내 흡연 여부와의 관련성에서는 성별, 연령, 결혼상태, 19세미만 가구원수, 교육수준, 가구소득, 주관적 건강수준, 주관적 스트레스, 하루 흡연량, 금연계획 여부, 금연캠페인 노출경험, 금연교육 경험 변수 모두에서 유의한 관련성을 나타냈다.

<Table 1 삽입>

**Logistic regression analysis with Smoke-Free indoor as the dependent variable**

가정 실내 흡연 관련 요인을 파악하기 위해 이분형 로지스틱 회귀분석을 시행하였으며, 분석 결과는 Table 2와 같다. 흡연자가 금연교육 경험이 있는 경우(OR=1.115) 결혼한 경우(이혼/사별/별거 OR=1.351; 기혼 OR=1.383), 19세 미만 가구원수가 많을수록(1명 OR=1.444; 2명 OR=1.711; 3명 이상 OR=1.583), 교육수준이 초등학교 졸업 이하에 비해 대학 졸업 이상일 때(OR=1.473), 월평균 가구소득이 높을수록(400-500만원 OR=1.089; 500만원 이상 OR=1.198), 주관적 건강수준이 좋을수록(보통 OR=1.171; 좋음 OR=1.271) 실내 흡연하지 않을 가능성이 높았다. 그에 반해 흡연자가 여성인 경우(OR=0.473), 연령이 많을수록(45-64세 OR=0.754; 65세 이상 OR=0.756), 스트레스가 높을수록(OR=0.900), 하루 흡연량이 많을수록(10-19개비 OR=0.670; 20-39개비 OR=0.479; 40개비 이상 OR=0.294), 금연계획 없을 경우(OR=0.705) 실내 흡연하지 않을 가능성이 낮은 것으로 나타났다.

<Table 2>

**DISCUSSION**

이 연구에서는 2017년 전국 지역사회건강조사 자료를 활용하여 가정 실내 흡연의 관련 요인을 파악하였다. 연구 결과 흡연자가 여성인 경우, 연령이 많을수록, 주관적 스트레스가 높을수록, 하루 흡연량이 많을수록, 금연 계획 없을 경우 실내 흡연하지 않을 가능성이 낮은 것으로 나타났으며, 흡연자가 결혼한 경우, 19세 미만 가구원 수가 많을수록, 교육수준이 초등학교 졸업 이하에 비해 대학 졸업 이상일 때, 월평균 가구소득이 높을수록, 주관적 건강수준이 좋을수록, 금연캠페인 노출 경험이 있는 경우, 금연교육 경험이 있는 경우 실내 흡연하지 않을 가능성이 높은 것으로 나타났다.

사회경제적 요인에서 교육수준이 초등학교 졸업 이하에 비해 대학 졸업 이상일 때, 월평균 가구소득이 높을수록 실내 흡연하지 않을 가능성이 높게 나타난 것은 높은 교육수준과 월 가구소득이 실내 금연과 관련이 있다는 선행연구 결과와 일치하였다 [17,18]. 저소득계층의 부모들의 간접흡연의 위험에 대한 지식이 낮고 이로 인해 간접흡연에 대한 태도와 대처가 상대적으로 미온적이기 때문에 가정 내 간접흡연이 더 많이 발생할 수 있다는 선행연구 결과로 설명할 수 있을 것이다 [26]. 이처럼 실내 흡연은 소득, 교육수준과 같은 사회경제적 요인에 영향을 받는 것으로 보고되고 있으므로 사회경제적 지위가 낮은 사람들의 실내 금연의지를 높일 수 있는 다양한 정책적 접근이 필요할 것이다 [27,28].

인구학적 요인에서 흡연자가 여성일 때 실내 흡연하지 않을 가능성이 낮았다. 이는 여성 흡연자의 주된 흡연 장소가 가정 실내 화장실, 가정 실내 베란다인 것으로 설명될 수 있을 것이다 [29-31]. 여성의 전자담배 이용률 또한 급격한 증가세를 보이고 있으며 궐련형 전자담배의 경우 연기가 수증기로 전환돼 냄새도 거의 나지 않아 실내 사용이 증가하고 있다는 점도 하나의 이유가 될 수 있을 것이다 [14,30]. 흡연자의 연령이 높을수록 실내 흡연하지 않을 가능성이 낮았는데, 이는 노인인 경우 가정 내 흡연에 대한 경각심이 젊은 사람들에 비해 낮아 가정 내 간접흡연이 발생할 수 있을 것으로 사료된다 [31]. 결혼 상태가 기혼이거나 기타일 때, 19세 미만 구성원 수가 많을수록 실내 흡연하지 않을 가능성이 높았다. 이는 결혼 상태가 기혼 또는 기타일 때, 자녀를 있을 가능성이 높으며 가정 내에서의 흡연을 자제할 가능성이 높을 것으로 판단된다 [23]. 특히 19세 미만 구성원의 경우 자녀일 가능성이 높으며, 가정의 어린 자녀가 있는 경우 가정 내 비흡연일 가능성이 높다는 기존 연구결과로 설명될 수 있을 것이다 [23,32,33]. 이는 아동 및 청소년에 대한 간접흡연의 악영향을 인지하고 있다는 점을 알 수 있으며, 향후 흡연자의 금연을 결심하는 하나의 원인이 될 수 있으므로 긍정적인 요소로 볼 수 있을 것이다. 건강관련 요인에서 주관적 건강수준이 좋을수록 실내 흡연하지 않을 가능성이 높게 나타났는데, 이는 주관적 건강수준이 건강에 긍정적인 영향을 미치는 건강 행위를 선택하는 데에 관련이 있다는 선행연구로 설명될 수 있을 것이다 [34,35]. 주관적 건강수준이 높다고 인식하는 계층에서 건강에 부정적인 영향을 미치는 흡연을 지양하고 실내 금연을 실천하는 것으로 추측된다. 반면 주관적 스트레스가 높을수록 실내 흡연하지 않을 가능성이 낮았으며, 이는 높은 스트레스로 인하여 흡연할 위험이 높아져 실내 금연을 어렵게 하는 요인으로 작용한 것으로 추측된다 [35]. 흡연관련 요인에서 하루 흡연량이 많을수록 실내 흡연하지 않을 가능성이 낮게 나타났으며, 이는 흡연 빈도가 낮을수록 실내에서 금연할 가능성이 높다는 선행연구와 일치하였다 [23,36,37]. 이는 흡연량이 적을수록 니코틴의존도가 낮아 금연 성공률이 높아진다는 선행연구 결과와 연관되어 있어, 흡연자의 흡연량이 적을수록 가정 내 다른 가구원들의 간접흡연 노출 가능성을 감소시킬 수 있음을 시사한다 [38]. 금연 계획은 금연 계획이 없음일 때 실내 흡연하지 않을 가능성이 낮게 나타났다. 이는 흡연자들의 금연 경향이 강할수록 실내 흡연할 가능성이 높다는 선행연구 결과로 설명될 수 있을 것이다 [39]. 금연교육 경험 여부에서 흡연자가 금연교육 경험이 있는 경우 실내 흡연하지 않을 가능성이 높았는데, 이는 금연교육 경험이 참여자의 금연 의지를 높여, 금연 행위를 긍정적으로 유도한다는 선행연구 결과와 일치하며 금연 교육이 금연의 동기부여로 작용한 결과로 생각된다 [40]. 2005년부터 전국 보건소(2019년 6월 기준 239개) 및 국가금연지원센터에서 제공하는 대표적인 교육 프로그램으로 가정에서의 흡연예방교육사업, 사업장 대상의 금연교육이 있다 [41]. 가정에서의 흡연예방교육사업은 ‘담배로부터 우리 아이를 보호하는 부모교육의 첫걸음’이라는 유아흡연예방교육 소책자(“담배로부터 우리 아이를 보호하는 부모교육의 첫걸음”)를 제공하며, 사업장 대상의 금연교육은 사업장의 금연 환경 조성을 위해 한국건강증진개발원과 지역사회(보건소, 지역금연지원센터)가 협업하여 제공하고 있다 [41,42]. 이처럼 다양한 금연 교육 프로그램을 제공되고 있지만, 시간적 접근성의 제약, 사업장 규모 및 특성에 따른 차별화된 교육 프로그램 등이 부재한 실정이다. 특히 소규모 사업장의 경우 건강증진사업의 사각지대로 남아 있는 실정이다 [40]. 따라서 흡연자들이 금연교육을 쉽게 접할 수 있도록 다양한 금연교육 자료를 제작하고 사업장 뿐만 아니라 주거지역 내 이동금연교실 등 찾아가는 금연지원서비스를 확대하는 방안이 필요할 것으로 보인다.

본 연구의 한계점은 다음과 같다. 첫째, 일개 연도만을 분석하였기 때문에 변수 간의 관련성은 확인할 수 있었으나, 인과관계를 파악하는 데는 어려움이 있다. 둘째, 이차 자료인 데이터의 한계로 인하여 연구대상자를 제외한 가정 내 흡연 인구 수, 가정 내 금연 규칙 여부와 같은 환경적 특성, 흡연자의 심리적 특성 등의 다양한 요인들을 고려하지 못하였다. 또한 가정 내 흡연자가 여러 명인 경우 가정 내 비흡연자가 간접흡연 시 조사대상자인 흡연자가 아닌 타 흡연자가 흡연한 경우도 분석에 포함되었을 수 있을 것이다.

이러한 한계점에도 불구하고 본 연구는 가정 실내 흡연과 관련된 다양한 요인들을 파악하였으며, 소득, 교육수준과 같은 사회경제적 요인과 가정 실내 흡연의 연관성을 확인하였다. 한국 성인을 대표할 수 있는 지역사회건강조사를 사용하여 사회경제적 요인을 중심으로 전국 성인의 가정 실내 흡연과 관련 있는 요인들을 파악하였다는 점에서 의의가 있다. 이를 통해 가정 실내 흡연 감소를 위한 금연 정책의 근거자료로 활용될 수 있을 것으로 기대된다.

**CONFLICT OF INTEREST**

The authors have no conflicts of interest to declare for this study.

**FUNDING**

None.

**ACKNOWLEDGEMENTS**

None.

**REFERENCES**

1. Witschi H, Joad JP, Pinkerton KE. The toxicology of environmental tobacco smoke. Annu Rev Pharmacol Toxicol 1997;37:29-52.

2. Okoli CT, Rayens MK, Wiggins AT, Ickes MJ, Butler KM, Hahn EJ. Secondhand tobacco smoke exposure and susceptibility to smoking, perceived addiction, and psychobehavioral symptoms among college students. J Am Coll Health 2016;64:96-103.

3. Kim J, Yoon JH, Kim J, Lee W, Kim YK, Lee S, et al. Factors associated with beliefs among adults in Korea about the health effects of thirdhand smoke on children. J Environ Health Sci 2018;44:90-97 (Korean).

4. Whitehead TP, Metayer C, Park JS, Does M, Buffler PA, Rappaport SM. Levels of nicotine in dust from homes of smokeless tobacco users. Nicotine Tob Res 2013;15:2045-2052.

5. Sleiman M, Gundel LA, Pankow JF, Jacob P 3rd, Singer BC, Destaillats H. Formation of carcinogens indoors by surface-mediated reactions of nicotine with nitrous acid, leading to potential thirdhand smoke hazards. Proc Natl Acad Sci U S A 2010;107:6576-6581.

6. Lee KY. Scientific evidences of thirdhand smoke. J Env Hlth Sci 2010;36:77~81 (Korean).

7. Centers for Disease Control and Prevention. 2014 Surgeon General's report: the health consequences of smoking—50 years of progress [cited 2020 Dec 19]. Available from: <https://www.cdc.gov/tobacco/data_statistics/sgr/50th-anniversary/index.htm>.

8. Padrón A, Galán I, García-Esquinas E, Fernández E, Ballbè M, Rodríguez-Artalejo F. Exposure to secondhand smoke in the home and mental health in children: a population-based study. Tob Control 2016;25:307-312.

9. World Health Organization. WHO report on the global tobacco epidemic 2017: monitoring tobacco use and prevention policies [cited 2020 Dec 18]. Available from: https://www.who.int/tobacco/global_report/2017/en/.

10. Centers for Disease Control and Prevention. Secondhand smoke: an unequal danger; 2015 [cited 2020 Dec 19]. Available from: https://www.cdc.gov/vitalsigns/pdf/2015-02-vitalsigns.pdf.

11. Kim BS, Kim A. The impacts of smoking bans on smoking in Korea. KDI J Econ Policy 2009;31:127-153 (Korean).

12. Gunter R, Szeto E, Jeong SH, Suh S, Waters AJ. Cigarette Smoking in South Korea: A Narrative Review. Korean J Fam Med. 2020;41(1):3-13.

13. You DH. E-cigarettes are no less harmful to second-hand smoke than regular cigarettes. ChosunMedia: 2019 Jul 5 [cited 2019 Aug 25]. Available from: <http://health.chosun.com/site/data/html_dir/2019/07/05/2019070500033.html> (Korean).

14. World Health Organization. 2014 Global progress report on the implementation of the WHO Framework Convention on Tobacco Control [cited 2020 Mar 1]. Available from: <https://www.who.int/fctc/reporting/2014globalprogressreport.pdf>.

15. Pizacani BA, Maher JE, Rohde K, Drach L, Stark MJ. Implementation of a smoke-free policy in subsidized multiunit housing: effects on smoking cessation and secondhand smoke exposure. Nicotine Tob Res 2012;14:1027-1034.

16. Cai L, Wu X, Goyal A, Han Y, Cui W, Xiao X, et al. Patterns and socioeconomic influences of tobacco exposure in tobacco cultivating rural areas of Yunnan Province, China. BMC Public Health 2012;12:842.

17. Abdullah AS, Hitchman SC, Driezen P, Nargis N, Quah AC, Fong GT. Socioeconomic differences in exposure to tobacco smoke pollution (TSP) in Bangladeshi households with children: findings from the International Tobacco Control (ITC) Bangladesh Survey. Int J Environ Res Public Health 2011;8:842-860.

18. Kang EJ. Smoking restriction rules and children's exposure to 49 SHS in homes. Health Welf Policy Forum 2007;132:59-70.

19. Jeong HM. A study on characteristics of current smokers and smoke free home [dissertation]. Seoul: Seoul National University; 2016 (Korean).

20. Korea Centers for Disease Control and Prevention Agency. Community Health Survey 2017. Cheongju: Korea Centers for Disease Control and Prevention; 2018, p. 4-7 (Korean).

21. Mills AL, Messer K, Gilpin EA, Pierce JP. The effect of smoke-free homes on adult smoking behavior: a review. Nicotine Tob Res 2009;11:1131-1141.

22. Borland R, Yong HH, Cummings KM, Hyland A, Anderson S, Fong GT. Determinants and consequences of smoke-free homes: findings from the International Tobacco Control (ITC) Four Country Survey. Tob Control 2006;15 Suppl 3(Suppl 3):iii42-iii50.

23. Jung KY, Yoo SH, Ma SH, Hong SH, Lee YS, Shim UY, et al. Inpatient smoking cessation program and its success rate for abstinence among Korean smokers. Korean J Fam Med 2009;30:503-510 (Korean).

24. Jun YW, Ji NJ, Lee WY. Factors associated with success of smoking cessation for 6 months at smoking-cessation clinic of public health center in urban area. Korean J Health Educ Promot 2008;25:25-38 (Korean).

25. Ryu SY, Shin JH, Kang MG, Park J. Factors associated with intention to quit smoking among male smokers in 13 communities in Honam region of Korea: 2010 Community Health survey. Korean J Health Educ Promot 2011;28:75-85 (Korean).

26. Min HY, Gwon GN. Exposure of preschoolers to secondhand smoke: parents' knowledge, attitudes and coping behavior. Korean J Child Stud 2005;26:101-112 (Korean).

27. Fernandez E, Schiaffino A, García M, Borràs JM. Widening social inequalities in smoking cessation in Spain, 1987-1997. J Epidemiol Community Health 2001;55:729-730.

28. Bobak M, Jarvis MJ, Skodova Z, Marmot M. Smoke intake among smokers is higher in lower socioeconomic groups. Tob Control 2000;9:310-312.

29. Agrawal D, Aggarwal AK, Goel S. Women exposed to second-hand smoke more at home than at workplace: An analysis of GATS Report, India, 2009- 10. J Family Med Prim Care 2015;4:293-7.

30. King AC, Smith LJ, McNamara PJ, Matthews AK, Fridberg DJ. Passive exposure to electronic cigarette (e-cigarette) use increases desire for combustible and e-cigarettes in young adult smokers. Tob Control 2015;24:501-504.

31. Comiford A, Garroutte E, Barbosa-Leiker C, Chen S, McDonell M. Prevalence and Indicators of Household Smoking Bans Among American Indians. J Community Health. 2018;43(4):746-755.

32. Farkas AJ, Gilpin EA, Distefan JM, Pierce JP. The effects of household and workplace smoking restrictions on quitting behaviours. Tob Control 1999;8:261-265.

33. Farkas AJ, Gilpin EA, White MM, Pierce JP. Association between household and workplace smoking restrictions and adolescent smoking. JAMA 2000;284:717-722.

34. Idler EL, Benyamini Y. Self-rated health and mortality: a review of twenty-seven community studies. J Health Soc Behav 1997;38:21-37.

35. Kim JD, Seo JH, Shin YJ, Kim CY. The factors associated with smoking behavior of low-income people. Health Soc Welf Rev2013;33:577-602 (Korean).

36. Hyland A, Higbee C, Travers MJ, Van Deusen A, Bansal-Travers M, King B, et al. Smoke-free homes and smoking cessation and relapse in a longitudinal population of adults. Nicotine Tob Res 2009;11:614-618.

37. Messer K, Mills AL, White MM, Pierce JP. The effect of smoke-free homes on smoking behavior in the U.S. Am J Prev Med 2008;35:210-216.

38. Lim KH, Idzwan MF, Sumarni MG, Kee CC, Amal NM, Lim KK, et al. Heaviness of smoking index, number of cigarettes smoked and the Fagerstrom test for nicotine dependence among adult male Malaysians. Asian Pac J Cancer Prev 2012;13:343-346.

39. Brinthaupt TM, Richard PL. Changing the self : philosophies, techniques, and experiences. Albany: State University of New York Press; 1994, p. 201-206.

40. Kim YH, Seo NS, Kang HY. Nicotine dependence, smoking-related attitude, and subjective norms across the stages of change for smoking cessation among adults smokers in a rural area. J Korean Acad Nurs 2006;36:1023-1032.

41. Korea Health Promotion Institute. National cessation service [cited 2020 Jan 10]. Available from: <https://nosmk.khealth.or.kr/nsk/ntcc/index.do> (Korean).

42. Korea Health Promotion Institute. Resolving smoking conflicts in the workplace...Create a tobacco-free working environment. [cited 2020 May 21]. Available from: <https://www.khealth.or.kr/board/view?linkId=999410&menuId=MENU00907> (Korean).
